# Lifetime risk of diabetes in metropolitan cities in India

**DOI:** 10.1007/s00125-020-05330-1

**Published:** 2020-11-23

**Authors:** Shammi Luhar, Dimple Kondal, Rebecca Jones, Ranjit M. Anjana, Shivani A. Patel, Sanjay Kinra, Lynda Clarke, Mohammed K. Ali, Dorairaj Prabhakaran, M. Masood Kadir, Nikhil Tandon, Viswanathan Mohan, K. M. Venkat Narayan

**Affiliations:** 1 Department of Public Health and Primary Care, University of Cambridge, Cambridge, UK; 2 Department of Population Health, London School of Hygiene and Tropical Medicine, London, UK; 3 Centre for Chronic Disease Control (CCDC), New Delhi, India; 4 Nutrition and Health Sciences, Laney Graduate School, Emory University, Atlanta, GA USA; 5 Madras Diabetes Research Foundation, Chennai, India; 6 Hubert Department of Global Health, Emory University, Atlanta, GA USA; 7 Department of Non-Communicable Disease Epidemiology, London School of Hygiene and Tropical Medicine, London, UK; 8 Public Health Foundation of India, Gurgaon, India; 9 Department of Community Health Sciences, Aga Khan University, Karachi, Pakistan; 10 Department of Endocrinology and Metabolism, All India Institute of Medical Sciences, New Delhi, India

**Keywords:** Body mass index, Diabetes, Diabetes-free life expectancy, India, Lifetime risk, Metropolitan cities, Urban

## Abstract

**Aims/hypothesis:**

We aimed to estimate the lifetime risk of diabetes and diabetes-free life expectancy in metropolitan cities in India among the population aged 20 years or more, and their variation by sex, age and BMI.

**Methods:**

A Markov simulation model was adopted to estimate age-, sex- and BMI-specific lifetime risk of developing diabetes and diabetes-free life expectancy. The main data inputs used were as follows: age-, sex- and BMI-specific incidence rates of diabetes in urban India taken from the Centre for Cardiometabolic Risk Reduction in South Asia (2010–2018); age-, sex- and urban-specific rates of mortality from period lifetables reported by the Government of India (2014); and prevalence of diabetes from the Indian Council for Medical Research INdia DIABetes study (2008–2015).

**Results:**

Lifetime risk (95% CI) of diabetes in 20-year-old men and women was 55.5 (51.6, 59.7)% and 64.6 (60.0, 69.5)%, respectively. Women generally had a higher lifetime risk across the lifespan. Remaining lifetime risk (95% CI) declined with age to 37.7 (30.1, 46.7)% at age 60 years among women and 27.5 (23.1, 32.4)% in men. Lifetime risk (95% CI) was highest among obese Indians: 86.0 (76.6, 91.5)% among 20-year-old women and 86.9 (75.4, 93.8)% among men. We identified considerably higher diabetes-free life expectancy at lower levels of BMI.

**Conclusions/interpretation:**

Lifetime risk of diabetes in metropolitan cities in India is alarming across the spectrum of weight and rises dramatically with higher BMI. Prevention of diabetes among metropolitan Indians of all ages is an urgent national priority, particularly given the rapid increase in urban obesogenic environments across the country.

**Figure:**
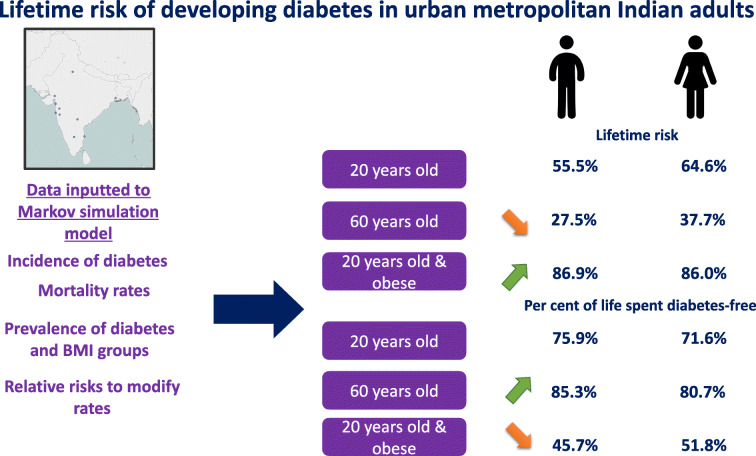
Graphical abstract

**Supplementary Information:**

The online version contains peer-reviewed but unedited supplementary material available at 10.1007/s00125-020-05330-1.

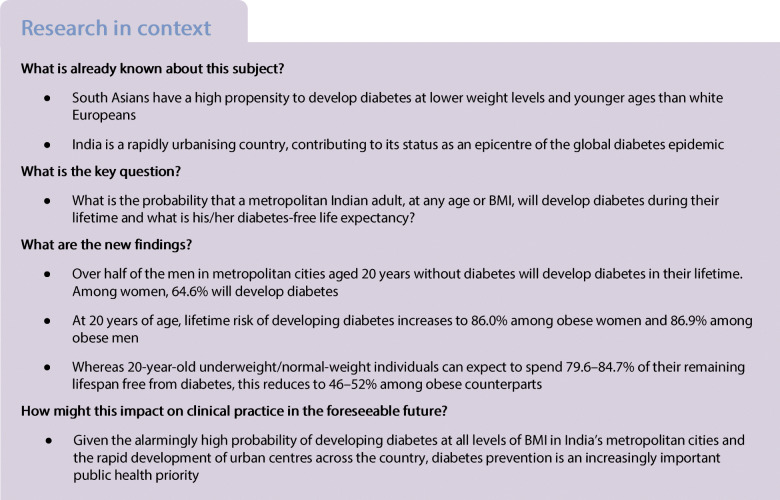



## Introduction

Diabetes is a major global public health problem currently affecting 463 million individuals and projected to affect 700 million by 2045 [1]. Estimates of prevalence suggest that the diabetes burden is increasing at a faster pace in low- to middle-income countries (LMICs) than in high-income countries (HICs). In India, 77 million adults currently have diabetes and this number is expected to almost double to 134 million by 2045 [1]. Whereas prevalence estimates are useful for expressing the overall burden of diabetes at a particular point in time, they do not inform future risk of developing this disease at the individual level. Lifetime risk provides an estimate of the cumulative probability of eventually developing diabetes over the course of life. It is a valuable estimate for effectively communicating diabetes risk to individuals, even at young ages, and is also a powerful tool with which to galvanise public health and policy responses. The few studies estimating the lifetime risk of developing diabetes are focused on HICs [2–4]. The population of India, while at the epicentre of the global diabetes epidemic, has a lower BMI distribution and lower overall life expectancy; Indian people also show a comparatively higher propensity to develop diabetes, both at younger ages and lower BMI levels [5], suggesting a substantially different epidemiology.

Studies from HICs such as the USA [2, 6], Australia [3] and the Netherlands [4] have found high lifetime risks of developing diabetes. For example, lifetime risk was 31.3% among 45-year-old individuals in the USA and 38.0% among those aged 25 years in the Netherlands. The lifetime risk among severely obese people in the USA was reported to be as high as ten times that of underweight individuals [2]. The combination of a high BMI distribution, high incidence of diabetes and high life expectancy drive a high overall lifetime risk in HICs. It is not clear, however, how the lifetime risk of developing diabetes may differ in LMICs wherein individuals may have a comparatively lower BMI and life expectancy.

In this study, we estimated the lifetime risk of developing diabetes in India’s metropolitan cities where an already large number of adults have diabetes and where there are rapid increases in population-level weight status and life expectancy [7]. Drawing on recent diabetes incidence data and on multiple Indian databases, we constructed a Markov model to estimate lifetime risk of diabetes in Indian metropolitan cities and also stratified by age, sex and BMI. We define metropolitan cities as per the Indian Census Commission’s definition: cities with over four million inhabitants (including Delhi, Mumbai, Kolkata, Chennai, Hyderabad, Bangalore, Ahmedabad, Pune, Surat and Nashik [8]; making up just over 5% of the population around the time of the last census [9]).

## Methods

Lifetime risk estimation required, as data inputs, age-, sex- and BMI-specific estimates of diabetes incidence and age-, sex- and BMI-specific rates of mortality by diabetes status. Differential risks of dying depending on diabetes and weight status, prevalence of diabetes and the prevalence of different BMI classes were used to modify population-level rates, making them specific to particular subgroups (i.e. BMI groups and individuals with and without diabetes).

This study was approved by the LSHTM Observational/Interventions Research Ethics Committee (ref. 17568).

### Incidence of diabetes

Incidence of diabetes was calculated using data from the India cArdiometabolic Risk Reduction in South Asia study (CARRS) cohort [10, 11], a metropolitan based cohort of 9812 participants with high retention and multiple points of follow-up, representative of Delhi and Chennai. CARRS was established in 2010–2011 and is under ongoing follow-up. Fasting venous plasma glucose (FPG) and HbA_1c_ data were collected at baseline in 2010–2011 as well as at the second and fourth follow-up assessments in 2013 and 2016, respectively. Age-specific population estimates of incidence were restricted to participants without diabetes at baseline and the annualised incidence rate was calculated at the second (2013) and fourth (2016) follow-up assessments. Incident diabetes was defined as occurring in an individual reporting no diabetes at baseline and baseline biochemical variables in the non-diabetes range (FPG <7 mmol/l and HbA_1c_< 48 mmol/mol [6.5%]) who subsequently recorded an FPG ≥7 mmol/l or HbA_1c_ ≥48 mmol/mol (6.5%) or reported diabetes treatment during follow-up. Incidence for the following age groups was available: 20–24 years; 25–34 years; 35–44 years; 45–54 years; 55–64 years; and ≥65 years. Using the RR of diabetes among different BMI groups, we modified the population rates to estimate incidence separately for the underweight/normal weight (BMI <25.0 kg/m^2^), overweight (BMI 25.0–29.9 kg/m^2^) and obese population (BMI ≥30.0 kg/m^2^) [12]. We report the incidence rates, in addition to the number of incident cases, from the CARRS study in the electronic supplementary material (ESM) Tables 1–5. Incidence rates by single years were subsequently extracted after fitting a cubic smoothing spline.

### Population mortality rates

Mortality rates by 5 year intervals were extracted from the latest Sample Registration System (SRS), which contains abridged lifetables for 2013–2017 by sex and urban residence [13]. The SRS provides conditional probabilities of death (lifetable notation = _*n*_*q*_*x*_), which we used to obtain age-specific mortality rates using an inversion of Chiang’s formula [14]. We obtained mortality rates by single years by fitting an age at death distribution to the 5 year mortality rates. Specifically, we used the Gompertz–Makeham law of mortality, which is made up of two separate components: an age-dependent part that states that the rate of mortality increases exponentially with age, representing the underlying mortality age pattern; and a component independent of age used to account for external causes of death, which may be especially high in early adulthood [14]. The mortality rates derived from the SRS lifetables are included in ESM Table 6 and the fit of the population mortality rates is shown in the ESM Figs 1–2. From this, we extracted predicted mortality rates by single years [15].

### Differential mortality rates by diabetes and BMI status

A meta-analysis of 35 published articles between 1990 and 2010 (220,689 individuals in HICs, mean follow-up 10.7 years) informed differential mortality rates in individuals with vs without diabetes. The study found 85% and 100% higher risk of all-cause mortality among men and women, respectively, for those with vs without type 2 diabetes [16]. We accounted for differential mortality among populations with different BMI status by applying RRs of dying by BMI group from a study that examined the association of BMI with mortality risk in urban Mumbai between 1991 and 2003 [17]. The study, comprising 148,173 individuals, reported 11% lower risk and 22% higher risk, of dying among overweight and obese men aged 35–59 years, respectively, compared with normal-weight men. Furthermore, they identified a 67% and 102% higher risk of death in underweight women and men, respectively, relative to normal-weight counterparts. Slightly attenuated RRs were found in individuals aged 60 years or more. For details of how differential mortality rates were calculated for the analysis, see ESM Methods.

### Age-specific prevalence of diabetes

Diabetes prevalence data were used to compartmentalise population rates of mortality into a set of rates specific to the population with and without diabetes. Age-specific diabetes prevalence was obtained from the Indian Council of Medical Research–INdia DIABetes (ICMR-INDIAB) study [18]. The ICMR-INDIAB study is the largest representative study with fasting glucose measures in India and includes data collected between 2008 and 2015 from 57,117 Indians from 14 states and one Union Territory. The ICMR-INDIAB study used 8 h fasting capillary blood glucose (CBG) measured by trained field workers to identify adults with previously undiagnosed diabetes. CBG is seen as a reasonable alternative to venous blood glucose in developing countries and has reasonable sensitivity (83.3–90.9%, depending on the diagnostic criteria) in India [19]. Using an 82.5 g oral glucose load (equivalent to 75 g of anhydrous glucose), 2 h post-load CBG was measured in individuals who did not self-report diabetes. Among those with self-reported diabetes, only fasting glucose was measured. Individuals with an 8 h CBG ≥7 mmol/l, 2 h CBG ≥12.2 mmol/l, or both, were categorised as having diabetes. Age was categorised into groups of 5 years.

### Age-specific prevalence of BMI categories

We used the proportion of the urban population in broad BMI categories to estimate weighted averages of lifetime risk for the whole population. The age- and sex-specific prevalences of ‘underweight/normal weight’ (BMI <25.0 kg/m^2^), ‘overweight’ (BMI 25.0–29.9 kg/m^2^) and ‘obesity’ (BMI ≥30.0 kg/m^2^) groups across the whole age range in urban Indians were obtained from a study that forecasted weight category prevalence in India using survey data [20]. The study obtained BMI group prevalence from the 2015–2016 National Family and Health Survey, which collected nationally representative health and demographic data on approximately 625,000 adult women aged 15–49 years and 93,000 adult men aged 15–54 years [21]. The Study on global AGEing and adult health (SAGE) wave 0 (2002–2004) and wave 1 (2007–2010) were used to obtain estimates of age-specific proportions of the urban population aged 50 years or more in broad BMI categories. SAGE is a nationally representative health and demographic study containing information on 2559 adults in wave 0 and approximately 3000 men and 3000 women in wave 1 [22].

### The model

We adopted a multistate Markov model to estimate the lifetime risk of diabetes [23]. A detailed and formal description of the methodology we adopted is described in a similar study [2]. The multistate model compartmentalises a population into three separate age-, sex- and BMI-specific groups: no diabetes; diabetes; and dead. The single-year incidence and mortality rates were inputted into a matrix of rates of which the exponential was subsequently taken to obtain a matrix of transition probabilities between the states based on starting age and state. This was performed separately by BMI category. These single-year transition probabilities, derived from the variables, simulated movements between the three mutually exclusive states (ESM Fig. 3) and the resultant transition matrix was used to generate measures of both lifetime risk of developing diabetes and diabetes-free life expectancy by single years. We were able to calculate total lifetime risk of developing diabetes as a weighted average of separate lifetime risk estimates for different BMI groups.

We performed multiple sensitivity analyses. First, we performed multiple simulations whereby we simultaneously selected random variables estimates from the distributions that informed their uncertainty. In total, 5000 simulations were carried out and the median lifetime risk estimate was reported as the final point estimate; the range of values at any age informed the uncertainty interval. Second, we supplemented our main lifetime risk estimates by BMI using South Asian-specific BMI cut-offs (BMI <23.0 kg/m^2^ for the underweight/normal-weight group, BMI ≥23.0 kg/m^2^ to <27.5 kg/m^2^ for the overweight group and BMI ≥27.5 kg/m^2^ for the obese group [24]). The higher positive relationship between body fat and BMI observed in South Asians when compared with white Europeans may make use of conventional BMI cut-offs inappropriate and lead to a relatively high disease risk at comparatively lower body weight [24]. Third, we tested the sensitivity of our model to the initial prevalence of diabetes (to modify diabetes and non-diabetes-specific mortality) using age-specific diabetes prevalence reported for the two Indian cities in the CARRS study, which used FPG rather than CBG used in the ICMR-INDIAB study, to determine diabetes status [25]. We also estimated the lifetime risk of developing diabetes using incidence rates that omitted HbA_1c_ in diagnosing diabetes. As individuals with incident diabetes can have normal FPG while having HbA_1c_ ≥48 mmol/mol (6.5%), this may have led us to overestimate the main lifetime risk estimates as they may represent a group with higher glucose tolerance than those satisfying both FPG and HbA_1c_-related diagnostic criteria [26]. In addition to BMI, we also examined the lifetime risk of diabetes by categories of waist circumference. Finally, we examined the effect on the total population lifetime risk of a change in the proportion of urban Indians classified as overweight or obese. Age-specific forecasts of the prevalence of excessive weight and obesity in urban India were extracted from a recent study [20]. All analyses were performed using the R (version 3.6.2) statistical software package (available for download at https://cran.r-project.org/bin/windows/base/old/3.6.2/).

## Results

### Main findings

Overall, the lifetime risk (95% CI) of developing diabetes among Indian metropolitans at age 20 years was 64.6 (95% CI 60, 69.5)% among women and 55.5 (51.6, 59.7)% among men. At ages 40 and 60 years, the remaining lifetime risk (95% CI) among women was 59.2 (52.4, 64.9)% and 37.7 (30.1, 46.7)%, respectively, and among men aged 40 and 60 years was 47.3 (42.4, 52.3)% and 27.5 (23.1, 32.4)%, respectively (Table 1).

**Table 1:** Lifetime risk of developing diabetes by global BMI cut points in Indian metropolitan cities

BMI group	Lifetime risk	
	Men	Women
Underweight/normal weight (BMI <25 kg/m^2^)		
Age 20 years	41.2 (36.7, 45.7)	51.6 (46, 58.4)
Age 40 years	34.5 (30.7, 38.8)	47.1 (40.5, 54.6)
Age 60 years	21.5 (16.7, 27.5)	30.7 (23.2, 40.1)
Overweight (BMI ≥25 kg/m^2^ to <30 kg/m^2^)		
Age 20 years	71.3 (64.6, 77.6)	71.0 (61.0, 80.6)
Age 40 years	63.8 (54.0, 72.1)	64.9 (54.1, 77.8)
Age 60 years	44.9 (32.2, 56.1)	45.9 (30.8, 65.6)
Obese (BMI ≥30 kg/m^2^)		
Age 20 years	86.9 (75.4, 93.8)	86.0 (76.6, 91.5)
Age 40 years	77.2 (56.7, 87.8)	79.1 (65.6, 88.8)
Age 60 years	55.9 (34.1, 74.6)	58.5 (32.9, 78.6)
Total population		
Age 20 years	55.5 (51.6, 59.7)	64.6 (60.0, 69.5)
Age 40 years	47.3 (42.4, 52.3)	59.2 (52.4, 64.9)
Age 60 years	27.5 (23.1, 32.4)	37.7 (30.1, 46.7)

Lifetime risk is presented as % (95% CI)

As expected, we found a higher age-specific lifetime risk of developing diabetes in higher BMI groups compared with lower ones. At age 20 years, the lifetime risk (95% CI) of developing diabetes among underweight/normal-weight men was 41.2 (36.7, 45.7)% compared with 71.3 (64.6, 77.6)% among overweight men and 86.9 (75.4, 93.8)% among obese men. Similarly, at age 20 years, the lifetime risk (95% CI) of developing diabetes among underweight/normal-weight women was 51.6 (46.0, 58.4)% compared with 71.0 (61.0, 80.6)% and 86.0 (76.6, 91.5)% among overweight and obese women, respectively.

Across BMI groups, the largest difference in lifetime risk of developing diabetes was between underweight/normal weight and overweight individuals. To illustrate, among urban men aged 20 years, the lifetime risk was higher by approximately 30 percentage points in the overweight group (71.3%) compared with the underweight/normal weight group (41.2%), whereas the lifetime risk was around 15 percentage points higher in obese men aged 20 years (86.9%) when compared with overweight counterparts.

Women at age 20 years can expect to live 38.9 of their remaining 54.4 years spent free of diabetes (71.6% of their remaining life) (Table 2). Similarly, men aged 20 years can expect to live 39.9 years of their remaining 52.6 years free of diabetes (75.9%). The remainder of life spent with diabetes varied considerably between BMI groups. Among underweight/normal weight women aged 20 years, on average, 43.4 years of an expected 54.5 remaining years (79.6%) were estimated to be diabetes-free, compared with 27.1 years of the remaining 52.4 years of life of obese women aged 20 years (51.8%).

**Table 2:** Percentage of remaining life spent with diabetes in Indian metropolitan cities by global BMI cut points at ages 20, 40 and 60

BMI group	Men			Women		
	Life expectancy (years)	Diabetes-free life expectancy (years)	% of remaining life without diabetes	Life expectancy (years)	Diabetes-free life expectancy (years)	% of remaining life without diabetes
Underweight/normal weight (BMI <25 kg/m^2^)						
Age 20 years	52.8	44.7	84.7	54.5	43.4	79.6
Age 40 years	34.4	28.8	83.5	35.7	27.6	77.2
Age 60 years	17.6	15.6	88.8	18.1	15.4	84.9
Overweight (BMI ≥25 kg/m^2^ to <30 kg/m^2^)						
Age 20 years	51.5	34.8	67.4	53.9	36.2	67.1
Age 40 years	33.6	22.2	66.2	35.2	23.0	65.3
Age 60 years	17.3	13.1	76.0	17.9	13.6	76.1
Obese (BMI ≥30 kg/m^2^)						
Age 20 years	49.3	22.6	45.7	52.4	27.1	51.8
Age 40 years	32.3	15.4	47.7	34.2	17.8	51.9
Age 60 years	17.3	11.1	64.1	17.6	11.6	65.9
Total population						
Age 20 years	52.6	39.9	75.9	54.4	38.9	71.6
Age 40 years	34.3	25.5	74.2	35.6	24.4	68.4
Age 60 years	17.7	15.1	85.3	18.2	14.7	80.7

### Sensitivity analysis findings

As expected, the overall lifetime risk of developing diabetes was attenuated when using South Asian-specific BMI cut-offs (Table 3). To illustrate, at age 20 years, the lifetime risk (95% CI) of developing diabetes was 37.4 (33.1, 44.3)% among underweight/normal-weight men, 63.6 (55.6, 71.9)% among overweight men and 83.3 (73, 91.1)% among obese men. For women aged 20 years, the lifetime risk (95% CI) was 46.8 (41.4, 53.0)% among underweight/normal-weight women, 67.6 (56.9, 75.2)% among overweight women and 80.3 (69.5, 88.3)% among obese women. We also assessed the effect of using age-specific prevalence of diabetes from CARRS, rather than the ICMR-INDIAB prevalence. The effect of this on the lifetime risk estimates was negligible (a change of 0–1 percentage points compared with initial estimates).

**Table 3:** Lifetime risk of developing diabetes by South Asian BMI cut points in Indian metropolitan cities

BMI group	Lifetime risk	
	Men	Women
Underweight/normal weight (BMI <23 kg/m^2^)		
Age 20 years	37.4 (33.1, 44.3)	46.8 (41.4, 53.0)
Age 40 years	32.0 (27.5, 39.2)	42.2 (36.0, 48.8)
Age 60 years	20.3 (15.2, 28.3)	27.0 (20.8, 34.6)
Overweight (BMI ≥23 kg/m^2^ to <27.5 kg/m^2^)		
Age 20 years	63.6 (55.6, 71.9)	67.6 (56.9, 75.2)
Age 40 years	55.7 (46.9, 64.8)	63.1 (51.3, 71.9)
Age 60 years	37.8 (25.5, 49.4)	44.2 (31.1, 56.8)
Obese (BMI ≥27.5 kg/m^2^)		
Age 20 years	83.3 (73.0, 91.1)	80.3 (69.5, 88.3)
Age 40 years	73.8 (60.8, 83.4)	72.1 (59.6, 83.7)
Age 60 years	53.5 (31.5, 71.8)	49.5 (29.3, 72.6)

Lifetime risk is presented as % (95% CI)

When using incidence rates that excluded HbA_1c_ in the determination of incident diabetes, we identified slightly different, although not notable, lifetime risk estimates (ESM Table 7). Overall lifetime risk (95% CI) at age 20 years among men and women, respectively, was 55.0 (51.1, 58.7)% and 59.2 (54.9, 63.7)% and decreased to 29.0 (25.0, 33.5)% and 32.8 (27.5, 37.7)% at age 60 years. Again, this masked considerable variation by BMI, whereby the lifetime risk (95% CI) at age 20 years in men and women, respectively, was as follows: 40.0 (36.4, 43.3)% and 40.6 (35.9, 45.2)% among underweight/normal-weight individuals; 69.9 (63.7, 75.1)% and 69.3 (62.8, 75.2)% among overweight individuals; and 88.9 (82.2, 93.3)% and 85.3 (77.3, 92.4)% among obese individuals.

We also estimated lifetime risks of developing diabetes according to waist circumference. At age 20 years, the lifetime risk (95% CI) was 71.0 (64.4, 80.9)% for men with a waist circumference ≥90 cm and 45.2 (40.9, 48.8)% for men with a waist circumference <90 cm (ESM Table 8). Among women aged 20 years, the lifetime risk (95% CI) was 75.5 (65.2, 84.2)% for those with waist circumference ≥80 cm and 44.5 (39.6, 49.4)% for those with waist circumference <80 cm.

Using the 2040 forecasted BMI group distribution in urban India, we estimated that the overall lifetime risk (95% CI) of developing diabetes among individuals aged 20 years would increase to 64.0 (60.0, 66.8)% and 77.1 (71.0, 81.7)% for men and women, respectively (ESM Table 9).

## Discussion

We found that in metropolitan India, the lifetime risk of developing diabetes among women and men without diabetes at age 20 years was 64.6% and 55.5%, respectively. The lifetime risk was consistently higher among women compared with men and declined with age. Two in five underweight/normal-weight 20-year-old men and over half of the underweight/normal-weight 20-year-old women were projected to develop diabetes, and the remaining risk increased to over eight in ten among obese individuals aged 20 years. We also found that obese metropolitans can expect to spend the greatest percentage of their remaining life with diabetes (around 50% from age 20 years).

These estimations relied on a number of assumptions. First, the model assumed that BMI-specific age-specific diabetes incidence will be constant throughout the lifetime of the cohort. This is a reasonable assumption given the lack of evidence as to how future BMI-specific diabetes incidence will develop. Second, we assumed that the transition to diabetes is permanent and that an individual cannot transition back to not having diabetes, an assumption we decided was reasonable given the relative rarity of spontaneous remission from diabetes. Third, we assumed that the RR of dying among those with diabetes relative to those without will remain constant into the future. The extent to which results changed through relaxation of this assumptions was negligible.

Our study has a number of strengths. Blood tests were used to identify diabetes and BMI was also objectively measured rather than using self-reports. Studies assessing the validity of self-reported diabetes have found considerable misclassification. For instance, one study found a sensitivity of self-reported diabetes in China was around 58.2% in urban areas and 35.0% in rural areas [27]. Low sensitivity of self-reported diabetes has the potential to underestimate the lifetime risk estimates of previous studies [2], potentially explaining differences between ours and previous findings from different settings. We also used data sources that are designed to be geographically representative to measure mortality rates, BMI and diabetes. As data on national-level incidence of diabetes are not available, we obtained incidence rates from a large well-characterised, well-retained and followed up, representative cohort in urban areas of North and South India, making our results both geographically representative and generalisable to the wider metropolitan population. Finally, we performed extensive sensitivity analyses, reporting a range of uncertainty around our estimates, providing supplementary lifetime risk estimates using South Asian-specific BMI cut-offs, accommodating for potential overestimation of diabetes incidence when using HbA_1c_ by using solely FPG and diabetes treatment to classify diabetes cases, and examining the sensitivity of the estimates to initial diabetes prevalence by using diabetes prevalence estimated in the CARRS cohort.

Our study also has a number of limitations. First, assuming that age-specific rates will prevail over the projection period may have led us to both underestimate lifetime risk of diabetes within a BMI group (as the individual BMI distribution within a broad BMI group shifts to the right) and underestimate the overall lifetime risk for the total population (as the prevalence of excessive weight and obesity has increased in recent decades [21], and will likely increase into the future). One study in the USA identified increases in overall population lifetime risk of 20 and 13 percentage points in men and women, respectively between 1985 and 2011 [6]. The latter concern was addressed in sensitivity analysis using forecasted prevalence of excessive weight and obesity in urban India. Second, life expectancy at all ages in India is expected to continue to increase over the coming years [28]. Longer life expectancies may increase the lifetime risk of diabetes, making our results possible underestimates. Third, the socioeconomic distribution of excessive weight and obesity is likely to continue to change. A recent study has documented an increasing proportion of the overweight and obese population coming from lower socioeconomic backgrounds [29] among whom the RR of dying compared with people from higher socioeconomic backgrounds is likely to be higher. This may have led us to potentially overestimate the lifetime risk for the obese people. Our results should be interpreted with some caution, especially given India’s considerable heterogeneity, somewhat limiting the generalisability of our findings to all of urban India. The results from this study relate to an average urban-dwelling individual in two highly urbanised areas; however, the extent of variation in lifetime risk and years of life lost to diabetes will depend on how much a particular individual differs from this average in regards their risk of diabetes [2, 30].

To our knowledge, this is the only study estimating the lifetime risk of diabetes in urban India. A comparison of our findings from those reported in HICs indicates an alarmingly high lifetime risk at every age in urban India. Recent data from the Netherlands indicates a 31.3% lifetime risk of diabetes among 45-year-olds; this contrasts with the reported 53.8% in urban Indian men and 55.4% in urban Indian women of the same age [4]. A study from the USA, using data from 2000–2011, reported a lifetime risk of diabetes of 40.2% among men and 39.6% among women aged 20 years. Our results are much closer to estimates of lifetime risk of diabetes among the black and Hispanic populations in the USA, groups considered at a higher risk of developing diabetes than the general population. A recent study found lifetime risks of developing diabetes in excess of 55.2% among black women and 51.5% in Hispanic women aged 20 years in the USA [6]. The higher lifetime risk among black and Hispanic women in the USA is driven by higher incidence when compared with white women. A study of diabetes incidence comparing urban Indian adults with black and white US residents reported consistently higher incidence in urban South Asians at all ages and BMI levels [11]. Overall, we posit that the considerably higher lifetime risk is driven by a relatively high predisposition to developing diabetes among Indians at both lower ages (up to a decade earlier) and lower BMIs when compared with white European populations [5].

On average, 70.1% of the remaining lifespan of the average woman and 74.0% of the remaining life of the average man without diabetes at the age of 20 years can be expected to be spent diabetes free; this proportion decreases considerably with increasing BMI. Attenuated results have been found in HICs; one study reported that Australians aged 25 years will spend 86% of their remaining years diabetes free [3], whereas a USA-based study found that women aged 18 years who were obese and severely obese could expect to spend around 80% and 65%, respectively, of their remaining life diabetes free [2].

The remarkably high lifetime risk of developing diabetes and the low diabetes-free life expectancy in urban India, especially for individuals with high BMI, implies that interventions targeting the incidence of diabetes may be of paramount importance moving forward. The importance is reinforced by the early onset of diabetes among South Asians, implying a greater proportion of life spent with diabetes and greater exposure to diabetes-related complications. Potentially effective interventions include high and sustained sugar-sweetened beverage taxation [31] or diabetes prevention programmes involving a combination of culturally tailored lifestyle interventions with a course of medication [32]. The success of any intervention will also require improved detection of individuals with diabetes and either impaired glucose tolerance or impaired fasting glucose, given that an estimated 57% of diabetes cases are undiagnosed in India [1]. Although in India the advancement from either impaired glucose tolerance or impaired fasting glucose to diabetes has been found to be comparatively faster than in other populations [7, 33], any level of insulin resistance adds to a very sobering picture of dysglycaemia and associated complications in a country where urban obesogenic environments are on the increase and public health infrastructure is strained. Additional strain is also likely to be exerted on individual families as personal expenditure on diabetes care can be as high as 34% of household income for some low-income urban families [34].

Metropolitan Indians at every age and BMI have an alarmingly high probability of developing diabetes compared with results from HICs, necessitating a strong demand for proactive efforts to prevent diabetes in metropolitan cities, given the rapid increase in urban obesogenic environments across the country [35]. Prevention of diabetes, especially in younger metropolitan Indians, should be a high priority for India. Future research into rural lifetime risks of diabetes, using diabetes incidence rates from such settings, is encouraged to provide a more complete snapshot of the overall lifetime risk of diabetes at the national level.

## Supplementary Information

ESM (PDF 428 kb)

## Data Availability

The publicly available SRS data have been deposited in the Office of the Registrar General & Census Commissioner repository (available at: http://censusindia.gov.in/vital_statistics/SRS_Based/SRS_Based.html). Data from the CARRS and the ICMR-INDIAB study can be accessed through liaison with the study’s principal investigators.

## Abbreviations

CARRS: cArdiometabolic Risk Reduction in South Asia study
CBG: Capillary blood glucose
FPG: Fasting venous plasma glucose
HIC: High-income country
ICMR-INDIAB: Indian Council of Medical Research–INdia DIABetes
LMIC: Low- to middle-income country
SAGE: Study on global AGEing and adult health
SRS: Sample Registration System
