# Supplementary material for: Lifetime risk of diabetes in metropolitan cities in India
Source: Diabetologia. 2020 Nov 23;64(3):521–9. doi: 10.1007/s00125-020-05330-1 (PMC7864818; doi:10.1007/s00125-020-05330-1)
Supplement: Supplementary file 1 — (PDF 428 kb) [file 125_2020_5330_MOESM1_ESM.pdf]

Electronic Supplementary Material

ESM Methods

Compartmentalising mortality rates into sub-categories of BMI and diabetes status

Separate mortality rates by BMI category were firstly calculated before further disaggregation by diabetes status.

The mortality rate among the underweight/normal weight population ($m_a^{u.n}$) was obtained in the following way:

$$m_a^{u.n} = \frac{m_a}{(P_a^{o.o} * r_a^{o.o:u.n}) - P_a^{o.o} + 1}$$

Where m_a refers to the age-specific population mortality rate in urban areas obtained from the Sample Registration System Abridged lifetables, $P_a^{o.o}$ is the age-specific prevalence of combined overweight and obesity in the urban population and $r_a^{o.o:u.n}$ is the relative risk of dying among the overweight and obese population relative to the underweight/normal weight population. This relative risk ($r_a^{o.o:u.n}$) was obtained in the following way:

$$r_a^{o.o:u.n} = \frac{(r_a^{ow:n} * \theta_a^{ow:oo}) + (r_a^{ob:n} * (1 - \theta_a^{ow:oo}))}{(r_a^{u:n} * \theta_a^{u:u.n}) + (1 * (1 - \theta_a^{u:u.n}))}$$

Whereby $\theta_a^{u:u.n}$ represents the proportion of the underweight/normal weight population who are underweight, and $r_a^{o.o:n}$ and $r_a^{u:n}$ represent the relative risk of dying among the combined overweight/obese population and underweight population, respectively, relative to the population in the normal BMI range.

Separate relative risks of dying among the overweight ($r_a^{ow:u.n}$) and obese ($r_a^{ob:u.n}$) population respectively, relative to the combined 'underweight/normal weight' population can be obtained in a similar way by changing the numerator to $r_a^{ow:n}$ and $r_a^{ob:n}$, respectively; both of which are derived from the literature (see main article). The separate mortality rates among the overweight (m_a^{ow}) and obese (m_a^{ob}) population are subsequently obtained using the following formulae:

$$m_a^{ow} = m_a^{u.n} * r_a^{ow:u.n}$$

$$m_a^{ob} = m_a^{u.n} * r_a^{ob:u.n}$$

The BMI-specific mortality rates are further compartmentalised into rates among individuals with and without diabetes using an adaptation of the above formulae. For instance, the mortality rate among individuals without(${}^{u.n}m_a^{ND}$), and with(${}^{u.n}m_a^{D}$), diabetes who are 'underweight/normal weight' is calculated as follows:

$$ {}^{u.n}m_a^{ND} = \frac{m_a^{u.n}}{(P_a^D * r_a^{D:ND}) - P_a^D + 1} $$

$$ {}^{u.n}m_a^{D} = {}^{u.n}m_a^{ND} * r_a^{D:ND} $$

Where $r_a^{D:ND}$ refers to the relative risk of dying among those who have diabetes relative to those who do not, and P_a^D refers to the age-specific prevalence of diabetes. Given the absence of diabetes prevalence by BMI, we assumed that the diabetes prevalence was the same in all BMI subcategories. Although diabetes prevalence can be strongly expected to increase with BMI, this did not change our model's lifetime risk output when tested due to the model's overall lack of sensitivity to mortality and initial diabetes prevalence.

ESM Tables

ESM Table 1. Age and BMI-specific incidence of diabetes with 95% confidence intervals (Ages 20-44)

		Incident cases	Person years	Incidence*	Lower	Upper
	Women					
Age (years)	20-24	7	1524.3	4.59	2.19	9.62
	25-34	56	6287.5	8.91	6.86	11.56
	35-44	126	6449.5	19.54	16.43	23.22
BMI (kg/m2) Global cut-offs	<=25	46	6754.7	6.81	5.11	9.08
	25-30	72	5043.7	14.28	11.35	17.95
	30+	71	2462.8	28.83	22.92	36.26
BMI (kg/m2) South Asian cut-offs	<=23	29	4742.3	6.12	4.25	8.79
	23-27.5	48	4752.7	10.10	7.62	13.38
	27.5+	112	4766.2	23.50	19.57	28.22
	Men					
Age (years)	20-24	7	1166.4	6.00	2.87	12.56
	25-34	39	3897.3	10.01	7.32	13.67
	35-44	80	5142.1	15.56	12.52	19.34
BMI (kg/m2) Global cut-offs	<=25	50	6546.0	7.64	5.80	10.07
	25-30	53	3037.8	17.45	13.36	22.78
	30+	23	622.0	36.97	24.76	55.22
BMI (kg/m2) South Asian cut-offs	<=23	26	4563.6	5.70	3.88	8.36
	23-27.5	51	3926.6	12.99	9.89	17.06
	27.5+	49	1715.6	28.56	19.66	35.00

*Rates are expressed per 1000 person years

ESM Table 2. Age and BMI-specific incidence of diabetes (Ages 45+)

		Incident cases	Person years	Incidence*	Lower	Upper
	Women					
Age (years)	45-54	85	3303.6	25.73	20.86	31.74
	55-64	38	1419.0	26.78	19.57	36.65
	≥65	12	501.4	23.93	13.68	41.86
BMI (kg/m2)	<=25	42	2149.7	19.54	14.48	26.36
Global cut-offs	25-30	51	1870.0	27.27	20.81	35.75
	30+	42	1204.3	34.87	25.91	46.94
BMI (kg/m2) South Asian cut-offs	<=23	21	1309.2	16.04	10.49	24.52
	23-27.5	51	1835.4	27.79	21.20	36.42
	27.5+	63	2079.5	30.30	23.76	38.64
	Men					
Age (years)	45-54	65	3240.4	20.06	15.77	25.52
	55-64	29	1389.3	20.87	14.56	29.92
	≥65	15	738.0	20.33	12.32	33.54
BMI (kg/m2)	<=25	47	3491.5	13.46	10.13	17.88
Global cut-offs	25-30	47	1474.3	31.88	24.06	42.24
	30+	15	401.9	37.33	22.72	61.32
BMI (kg/m2) South Asian cut-offs	<=23	30	2503.2	11.98	8.40	17.10
	23-27.5	47	1926.9	24.39	18.39	32.35
	27.5+	32	937.6	34.13	24.28	47.98

*Rates are expressed per 1000 person years

ESM Table 3. Age and BMI-specific incidence of diabetes (Ages 20-44) based solely on FPG and self-report

		Incident cases	Person years	Incidence*	Lower	Upper
	Women					
Age (Years)	20-24	5	1556	3.21	1.34	7.71
	25-34	51	6625	7.70	5.86	10.12
	35-44	123	7174	17.15	14.39	20.43
BMI (kg/m2) Global cut-offs	<=25	32	7013	4.56	3.23	6.45
	25-30	74	5439	13.61	10.85	17.06
	30+	73	2903	25.15	20.05	31.54
	Men					
Age (Years)	20-24	2	1226	1.63	0.41	6.52
	25-34	38	4176	9.10	6.63	12.49
	35-44	92	5709	16.11	13.16	19.74
BMI (kg/m2) Global cut-offs	<=25	41	6869	5.97	4.40	8.10
	25-30	58	3448	16.82	13.03	21.71
	30+	33	794	41.56	29.76	58.04

*Rates are expressed per 1000 person years

ESM Table 4. Age and BMI-specific incidence of diabetes (Ages 45+) based solely on FPG and self-report

		Incident cases	Person years	Incidence*	Lower	Upper
	Women					
Age (Years)	45-54	120	4334	27.69	23.21	33.03
	55-64	48	1904	25.21	19.07	33.33
	≥65	20	715	27.97	18.16	43.09
BMI (kg/m2) Global cut-offs	<=25	35	2522	13.88	9.99	19.28
	25-30	72	2522	28.55	22.74	35.85
	30+	81	1909	42.43	34.29	52.51
	Men					
Age (Years)	45-54	84	3788	22.18	17.95	27.40
	55-64	39	1803	21.63	15.86	29.50
	≥65	25	1060	23.58	16.01	34.74
BMI (kg/m2) Global cut-offs	<=25	56	4027	13.91	10.72	18.04
	25-30	61	1970	30.96	24.19	39.64
	30+	31	654	47.40	33.62	66.83

*Rates are expressed per 1000 person years

ESM Table 5. Age and Waist Circumference-specific incidence of diabetes based solely on FPG and self-report

Age	Waist circumference	Incidence* ¶	Lower	Upper
		Women		
20-44	<80cm	6.13	4.58	8.20
45+	<80cm	14.75	10.28	21.17
20-44	>=80cm	19.19	16.41	22.44
45+	>=80cm	31.08	25.97	37.21
		Men		
Age	Waist circumference	Point est	Lower	Upper
20-44	<90cm	8.25	6.44	10.57
45+	<90cm	15.03	11.42	19.79
20-44	>=90cm	20.44	16.36	25.53
45+	>=90cm	28.15	22.38	35.40

*Age-specific population-level rates are same as in ESM Tables 1 and 2

¶ Rates are expressed per 1000 person years

ESM Table 6. Age-specific mortality rates (annualised rates per individual) for men and women from the 2015 Sample Registration system in Urban India

Age	Men	Women
0-1	0.02465	0.02824
1-4	0.00068	0.00060
5-9	0.00049	0.00050
10-14	0.00049	0.00044
15-19	0.00074	0.00073
20-24	0.00115	0.00096
25-29	0.00133	0.00086
30-34	0.00185	0.00098
35-39	0.00275	0.00143
40-44	0.00351	0.00192
45-49	0.00511	0.00280
50-54	0.00709	0.00512
55-59	0.01072	0.00793
60-64	0.01420	0.01151
65-69	0.02084	0.01826
70-74	0.02902	0.02606
75-79	0.03976	0.03657
80-84	0.05187	0.04959
85+	0.19404	0.18867

ESM Table 7. Lifetime risk of diabetes (%) at ages 20, 40 and 60 years by global BMI cut points in Indian metropolitan cities (diagnostic criteria of incident case excludes use of HbA1c)

		Men	Women
BMI group	Age (years)	Lifetime risk (%)	Lifetime risk (%)
Underweight/ Normal weight ($BMI<25kg/m^2$)	20<br>40<br>60	40.0 (36.4-43.3)<br>35.7 (32.2-39.8)<br>23.2 (19-28.6)	40.6 (35.9-45.2)<br>36.9 (32-41.4)<br>23.9 (19.1-28.7)
Overweight ($BMI>=25$ & $<30kg/m^2$)	20<br>40<br>60	69.9 (63.7-75.1)<br>63.4 (55.7-71.1)<br>44.2 (34.1-56.3)	69.3 (62.8-75.2)<br>63 (53.2-70.6)<br>43.6 (33.2-56.1)
Obese ($BMI>=30kg/m^2$)	20<br>40<br>60	88.9 (82.2-93.3)<br>82.1 (70.2-89)<br>62.1 (41.4-78.7)	85.3 (77.3-92.4)<br>79.3 (66.1-88.1)<br>59.0 (43-73.9)
Total Population	20<br>40<br>60	55.0 (51.1-58.7)<br>48.7 (44.5-52.9)<br>29 (25-33.5)	59.2 (54.9-63.7)<br>53.9 (48.9-58.6)<br>32.8 (27.5-37.7)

ESM Table 8. Lifetime risk of diabetes (%) at ages 20, 40 and 60 years by WC cut points in Indian metropolitan cities

		Men	Women
Waist circumference	Age (years)	Lifetime risk (%)	Lifetime risk (%)
WC < 90cm (men); WC<80cm (women)*	20<br>40<br>60	45.2 (40.9-48.8)<br>38.0 (34-42.4)<br>23.3 (19-28.4)	44.5 (39.6-49.4)<br>39.5 (34-44.7)<br>25.0 (19.3-32.4)
WC>= 90cm (men); WC>=80cm (women)	20<br>40<br>60	71.0 (64.4-80.9)<br>60.5 (51-72.2)<br>39.4 (29-52.3)	75.5 (65.2-84.2)<br>67.8 (54.4-79.1)<br>46.4 (31.3-62.4)

* Relative risk of dying among those classified as centrally obese relative to those classified as not centrally obese extracted from Cerhan et al (2014) [1]

ESM Table 9. Lifetime risk of diabetes in the total population using the 2040 forecasted BMI group distribution from Urban India

		Men	Women
BMI group*	Age (years)	Lifetime risk (%)	Lifetime risk (%)
Total Population	20	64.0 (60.0-66.8)	77.1 (71.0-81.7)
	40	57.9 (53.5-60.8)	71.1 (63.5-77.3)
	60	37.6 (33.5-41.7)	49.8 (38.9-63.0)

* Forecasted proportion of the population in the BMI categories extracted from Luhar et al (2020)[2]

ESM Figures

ESM Figure 1. 2015 age-specific log mortality rates (annualised per individual) in Urban India from the Sample Registration System and fitted rates (Women)

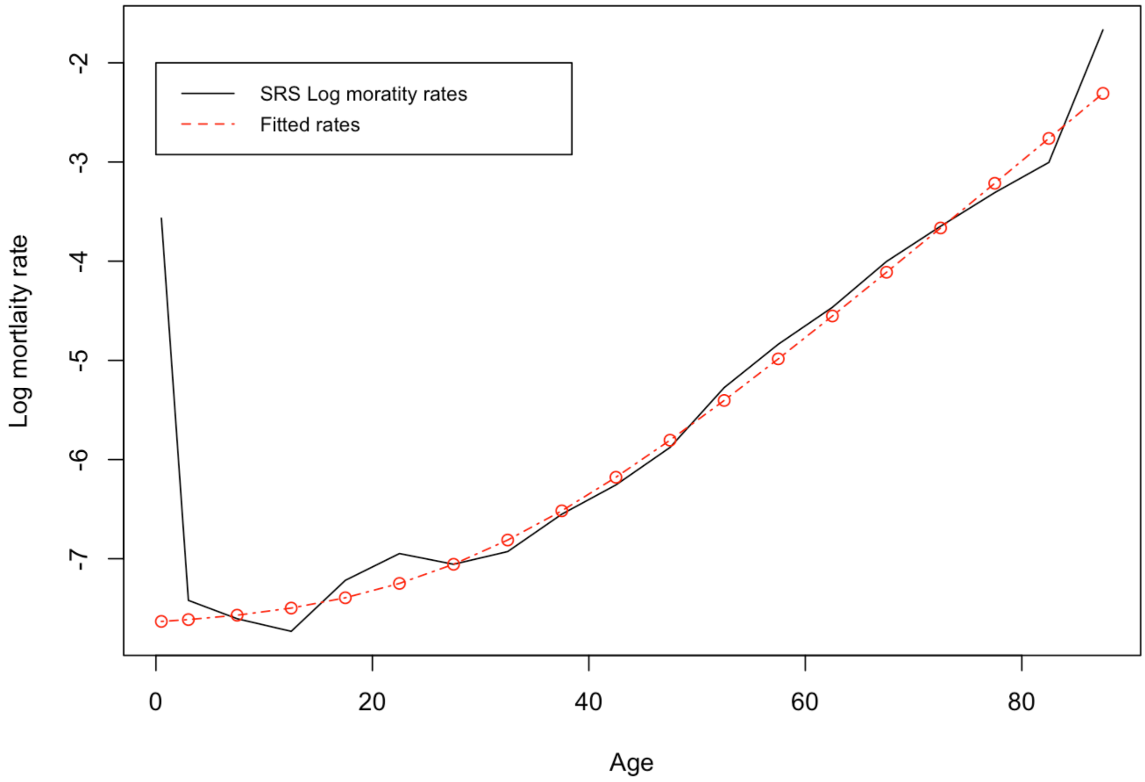

ESM Figure 2. 2015 age-specific log mortality rates (annualised per individual) in Urban India from the Sample Registration System and fitted rates (Women)

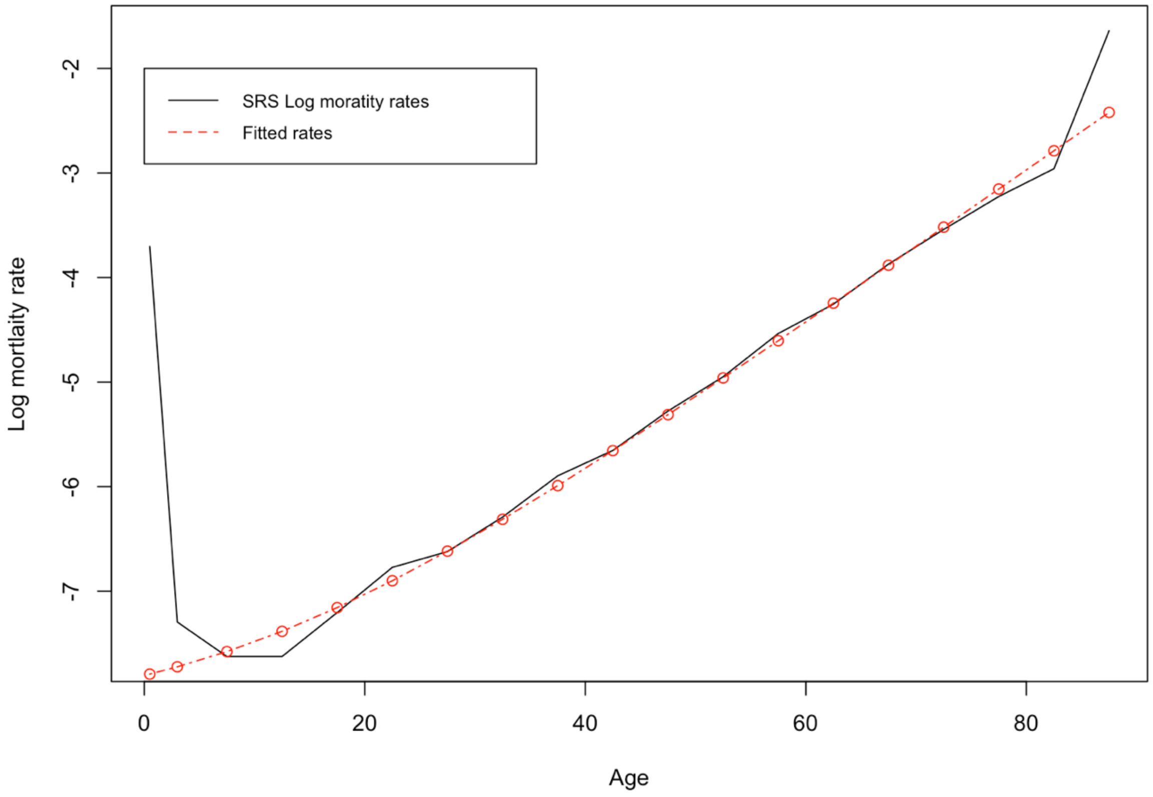

ESM Figure 3. Model used to estimate the lifetime risk of diabetes in Indian metropolitan cities*

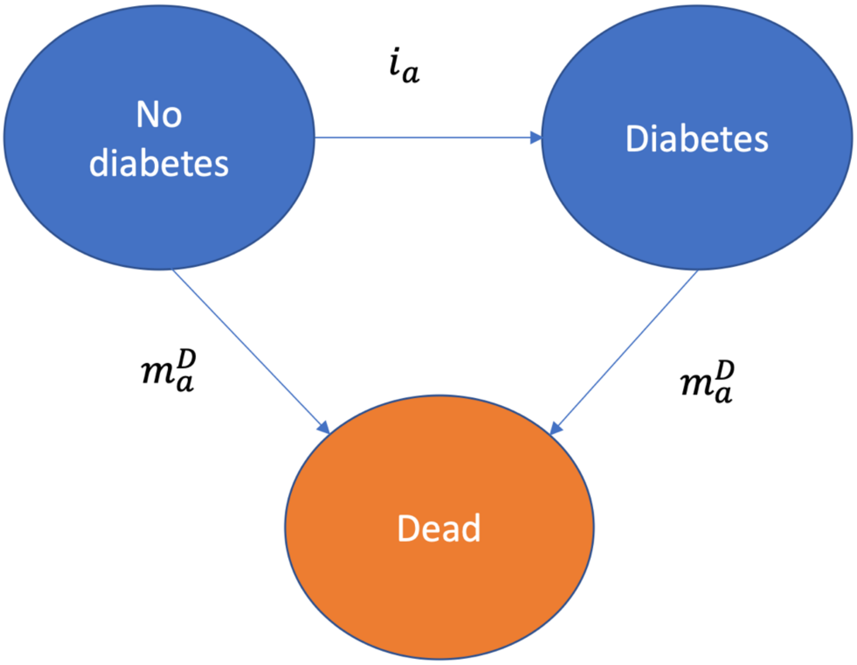

* i_a refers to the age-specific rate of incidence at age a; m_a^D and m_a^{ND} refer to the diabetes and non-diabetes-specific mortality rates at age a.

References

1. Cerhan, J. R., Moore, S. C., Jacobs, E. J., Kitahara, C. M., Rosenberg, P. S., Adami, H. O., *et al.* (2014). A pooled analysis of waist circumference and mortality in 650,000 adults. *Mayo Clin. Proc.* **89**, 335–345
2. Luhar, S., Timæus, I. M., Jones, R., Cunningham, S., Patel, S. A., Kinra, S., *et al.* (2020). Forecasting the prevalence of overweight and obesity in India to 2040. *PLoS One* **15**, p.e0229438
